# Infantile spasms: review of the literature and personal experience

**DOI:** 10.1186/1824-7288-36-15

**Published:** 2010-02-08

**Authors:** Alberto Fois

**Affiliations:** 1 Institute of Clinical Pediatrics, University of Siena, Siena, Italy

## Abstract

This epileptic disorder has become a classic topic for neuropediatricians and the interest is documented by the large number of publications on this subject.

The relative frequency among the epileptic syndromes is an another reason why not only neuropediatricians but also general pediatricians must be fully informed about diagnostic, clinical, imaging and genetic aspects.

Early diagnosis is of paramount importance in order to obtain even complete results in patients with so called idiopathic situations. A number of problems are still to be solved. There is no agreement on the type and the schedule of treatment. A common denominator about this problem is not jet available even if some advances in this regard have been accomplished. Of paramount importance is an accurate clinical and laboratory examination as a prerequisite regarding prognosis and results of therapy in every single case.

However, even if more than 170 years have elapsed since the first communication of dr. West on the peculiar syndrome that his child was suffering of, the interest of scientists on this subject has now been enriched and rewarded.

## Introduction

This epileptic disorder of infancy or early childhood was first described by Dr. William James West in 1841 in his son [1].

It is characterized by the triad of infantile spasms, hypsarrhythmia, and retardation. The EEG pattern (hypsarrhythmia) was illustrated by Gibbs [2]. The description of Dr. West is exhaustive: the spasms begun when his son was 4 months old and are described accurately. "The bowing and relaxing of the head and trunk would be repeated alternatively at intervals of few seconds and from ten to twenty or more times at each attack that would continue not more than two or three minutes ...sometimes two, three or more attacks in the day. The child appeared frightened and screamed.... he neither possessed the intellectual vivacity or the power of holding himself upright or using his limbs, and his head falls without support." Dr. West assumed that spasms were caused by an irritation of the nervous system, perhaps caused by teething. The used remedies were no avail. The patient died in an institution at the age of 20 years [3].

Infantile spasms (IS) is "one of the catastrophic childhood epilepsies" due to the difficulty of controlling seizures and the association with mental retardation. Early diagnosis with a careful diagnostic evaluation and proper therapy can obtain a normal development or a much improved situation in some cases [4].

This syndrome is also referred in literature as massive spasms, Salaam tics, infantile myoclonic seizures or Blitz-Nick-Salaam Krämpfe. It has been classified in the category of generalized seizures with specific EEG characteristics. Focal seizures as well as focal lesions can also be present [5].

IS have been classified as idiopathic when another neurologic disorder is not identified, cryptogenic when a possible etiology is suspected but not identified and symptomatic when a definite etiology can be demonstrated. From this point of view it is important to distinguish if patients were neurologically normal before the onset of symptoms from those in which this is not the case. It must be noted that the terms idiopathic or cryptogenic are used by some authors interchangeably when symptoms developed in a previously normal child.

## Epidemiology

The incidence of IS has been estimated to be between 1:4000 to 1:6000 live births [6] and has not substantially changed. In the report of Riikonen the overall incidence of IS was 30,7/100.000 live births [7]. Etiologic classification was symptomatic in 68% cryptogenic in 24% and idiopathic in 8% of children. There can be however a marked variability in annual and five years incidence rates [8].

## Evolution

Spasms usually appear in the first 2 years of life with a peak between 4 and 6 months and sometimes continue until adolescence. In cases that do not respond to therapy they are substituted by different types of seizures. The disorder can evolve in a Lennox-Gastaut syndrome: this possibility can favour the hypothesis that this happens because the syndromes manifestation are age related [9].

## Relationship with Other Epileptic Syndromes

Early myoclonic encephalopathy (EME) and Ohtahara syndrome are currently listed as two separate syndromes in the classification of epilepsy.

Patients with Ohtahara syndrome have prevalently tonic seizure that frequently evolve in infantile spasms: the prognosis is often worst than in patients with EME.

However there is an overlap in the etiologies. Tonic seizures are considered a manifestation of brainstem dysfunction and is possible that they are more prominent in the Ohtahara syndrome [10].

## Etiology

A number of pathologies has been documented as associated factor for IS. These are indicated in Appendix 1.

Metabolic disorders have been implicated. To list only the most recent: d-glyceric aciduria [11], Menkes disease [12], mitochondrial diseases [13,14], pyruvate dehydrogenase E1 alpha subunit deficiency [15], phenylketonuria [16], hypoglycaemia [17], Alexander disease [18], CDG deficient syndrome, biotinidase deficiency [19], Leigh syndrome [20], methylmalonic aciduria [21], propionic acidemia [22], vitamin B12 deficiency [23], d-bifunctional protein deficiency [24], SCAD deficiency [25], Hurler syndrome [26].

Malformations such as microcephaly, lissencephaly and tetralogy of Fallot [27] and malformation of cortical development (MCDS) can cause IS. In this last possibility the MRI can be normal but functional neuroimaging can be indicative [28]. In these cases the hypsarrhythmia can be asymmetric. Familiar periventricular heterotopia with mutation in filamin A (FLNA) gene, an X-linked dominant disorder, can cause West syndrome in males [29,30]. Tuberous sclerosis complex (TSC) is a frequent cause of symptomatic IS [31]. Gyration abnormalities [32] polimicrogiria [33] and [34] are also associated.

Dysmorphogenetic sindromes are also described together with IS. Aicardi syndrome is an X-linked dominant condition characterized by IS, agenesis of corpus callosum and chorioretinal lacunae. It occurs only in individuals with two X chromosomes and is not familial. The brain malformation is complex with cortical migration abnormalities heterotopias microgyria or pachigiria and subependimal cortical heterotopias, often cystic formations and sometimes choroids plexus papillomas. The eye anomalies, often feature a coloboma in addition to the lacunae, are common. Since it has been reported in two boys with XXY, complement, an X-linked gene lethal in early pregnancy for males is supposed [35,36]. Kabuki syndrome with multiple malformation and peculiar face which resembles a Kabuki actor [37]. Norrie disease, a rare X-linked recessive disorder with congenital blindness [38], Schinzel-Giedion syndrome characterized by a midfacial retraction, kidney and urinary malformations, multiple skeletal abnormalities and neurodegeneration [39], Sotos syndrome (cerebral gigantism) [40] and Williams syndrome with craniosynostosis [41] have also been found to be associated.

Another possible association is PEHO syndrome a progressive encephalopathy with edema, hypsarrhytmia and optic atrophy. It is a rare neurodegenerative disorder: most patients are of Finnish descent. There is marked cerebellar atrophy and infantile spasms [42,43]. IS can also be associated in DEND syndrome, characterized by developmental delay, epilepsy and neonatal diabetes [44].

IS can have an infectious origin which can be supposed or demonstrated, in particular with exanthema subitum. Cytomegalovirus has been also implicated [45,46]. These cases can undergo spontaneous remission.

Cerebral tuberculomas have also been reported [47,48]. These observations together with observed immunologic abnormalities [49] and an increase of interleukin -6 levels in the cerebrospinal fluid [50] are in favour of an immune pathogenesis.

The possible relationship between vaccinations and West syndrome hypotesized for long time, has been recently ruled out [51].

Neoplasms such as leptomeningeal angiomatosis [52] and basal ganglia glioma have been implicated [53].

Destructive lesions (encephalomalacia) Sturge-Weber syndrome and neoplasm can cause IS but the most common neuropathologic finding is cortical dysplasia (CT). This last finding underscores the similarity of IS in tuberous sclerosis [54].

There is a temporal latency between a lesional event and the onset of ISS which often cannot be determined. The interval between brain injury and the onset of infantile spasms ranges from 6 weeks to 2 months and a similar interval occurs in children with perinatal cerebral infarction. This time interval is against the close temporal association between immunization and onset of IS [55].

Genetic errors such as Down's syndrome [56], trisomy 4 p [57], cri du chat syndrome [58], terminal 1p36 deletion [59] can cause IS. Changes at molecular level such as sodium channel mutations [60] have now been found.

In the large majority of cases West syndrome is not familiar but sometimes can be transmitted as X-linked disorder. In this case the mutation is related to ARX homeobox gene which is a homolog of the drosophila gene Aristaless (OMIM 300382) located on Xp 22.13. It contains 5 exons and encodes a protein which may regulate brain development [61] The most common mutation in ARX is a 24 bp duplication in exon 2 resulting in an expansion of the polyalanine tract. The Aristaless-paired homeobox, is a highly conserved octapeptide expressed in the brain as well as in other tissues. Many disease phenotypes have been associated with this gene. Among them are severe dystonia and quadriparesis without neuro-imaging findings of perinatal injury but small areas of abnormally increased signal intensity in the putamina, spasticity, intellectual disability and myoclonic epilepsy. The ARX mutations possibly causes intranuclear aggregation of mutant ARX protein and cell death in neurons [62-64]. Mutation in another gene can cause severe developmental disorder with infantile spasms.

Since ARX is also associated to X-linked mental retardation (MR) and other epileptic syndromes, this finding suggest the existence of a relationship between MR and epilepsy at molecular level [65].

Mutations in ARX gene have been found also in autism and dystonia but also in sporadic cryptogenetic infantile spasms in X-linked lissencephaly with abnormal genitalia (XLAG), agenesis of corpus callosum and midbrain malformations (ACC) [66-68].

In two female patients with de novo balanced autosome translocations, the serine-threonine kinase 9 (STK9) gene was disrupted. This gene maps distal to ARX in Xp 22.3 region. The phenotype of these two sisters was identical consisting in early onset IS, profound global developmental arrest, hypsarrhythmia ad severe mental retardation. This suggest that lack of STK9 functional protein causes severe X linked IS (ISSX) and moreover indicate that STK9 is a second locus for this disorder [69]. More recently this gene has been demonstrated to be the same of CDKL5 and therefore it is related to this encephalopathy. Polyalanine tract expansion (33 bp duplication) has been found also in a patient with Ohtahara syndrome [70].

## Pathogenesis

It was been suggested that IS may result from a particular temporal desynchronization of two or more central nervous system developmental processes resulting in a specific disturbance of brain function, postulated to be dependent on an unbalanced maturation pattern. This disturbed function could result from multiple causative factors and therefore be associated with a variety of different structural and/or biochemical abnormalities compatible with the observed multiplicity of etiological associations [71]. Since IS are often associated with cortical abnormalities Chugani proposes an hypothesis which considers the involvement of a cortical and subcortical mechanism [72].

As already mentioned the recent consensus for pertussis vaccination (a debated issue for more 50 years) is that the risk of vaccine induced encephalopathy and/or epilepsy, if it exist at all, is extremely low [50].

## Clinical Findings

The most typical manifestations are the spasms. They are muscular contractions lasting 1-2 seconds. They are slower than a myoclonic jerk but more rapid of a tonic seizure. Spasms can be synchronized in the EEG with a fast head nods or with violent flexion of trunk, arms and legs. Brief spasms can be easily misinterpreted by parents or physician and, especially when consisting of flexion of inferior limbs on the abdomen, considered as "colics". Spasms can be of the flexor or extension or mixed flexor extension types of the neck, trunk, arms and legs. When asymmetric they are and usually present in symptomatic cases and temporally associated with partial or generalized seizures. Autonomic symptom such as skin flushing, sweating, pupillary dilatation or changes in respiratory or heart rate can be present [73,74].

Spasms often appear in cluster, their number varying conspicuously, also during the sleep or the awakening. Irritability or crying is frequent during or after the spasms. In the idiopathic cases the child developmental level at the beginning of symptoms may appear normal. However, already after few days the parents may notice a slower attention or irritability. In patients with an identifiable cause different neurologic abnormalities or retardation are present.

## EEG Findings

**Figure 1:**
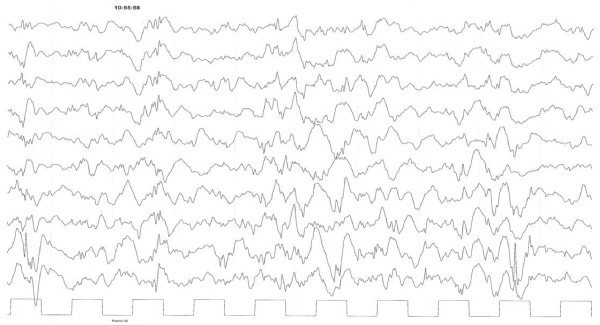
**Spontaneous sleep Eight month old male baby**. Hypsarrhythmia. Continuous diffuse irregular slow waves mixed with multifocal spikes. Absence of normal sleep features.

Marked abnormalities of the EEG are usually present. In the idiopathic cases the initial EEG, if the onset of symptoms is very recent, can be normal or borderline. In these cases a repeat EEG after 7-10 days is necessary. The most typical findings is the so called hypsarrhytmia [75] (Fig 1).

It consist in a mixed pattern of high voltage slow waves mixed with spikes or polyspikes. These abnormalities are continuous or almost continuous. Some form of modified hypsarrhythmia have been described with associated focal discharges, interhemispheric asynchrony or periodic attenuation of the discharges. The EEG equivalent of the myoclonic jerks is a brief burst of spike and waves or polyspikes activity during a tonic seizure. The normal sleep pattern is absent. Sometimes sleep spindles may be noted. They usually reappear when the result of therapy is favourable. The hypsarrhythmic pattern is very useful in differentiating West syndrome from others non epileptic attacks.

## Differential Diagnosis

In this regard other epileptic and nonepileptic manifestations must be considered. Spasms are different from myoclonic jerks and tonic seizures. Polygraphy is very useful for the differentiation. Myoclonic jerks are a rapid muscular contractions lasting less than 200 milliseconds with an EEG correlate of a diffuse fast spikes and waves or polyspike discharges. Tonic seizures are prolonged muscular contractions with progressive intensity and EEG correlate of fast spikes. As already mentioned the EEG finding in spasms is a spike and wave discharge with a prominence of the slow waves. For the differentiation is important the type and duration of the muscular discharge. Epileptic spasms with late onset are described: they could represent an intermediary type of epileptic encephalopathy between West and Lennox-Gastaut syndrome [76].

Epileptic spasms even in clusters can be present without EEG findings of typical or modified hypsarrhythmia with or without focal paroxysmal discharges on the EEG and normal metabolic and imaging findings. It is not clear if they must be considered a variant of West syndrome [77]. Benign spasms are tonic contractions while awake or asleep. The EEG is normal [78].

IS can evolve in Lennox-Gastaut syndrome. Ohtahara syndrome (OS) can develop in IS. It is the earliest form of age dependent epileptic encephalopathy:it is characterized by frequent tonic spasms which begin in the first months of life and a suppression burst pattern in the EEG. The cardinal seizure type in OS is tonic seizures occurring in cluster, either in the waking or the sleeping state. Focal motor seizures and hemiconvulsions are present in about half of the cases. Seizure are often intractable. The differentiation from the IS is useful but not so important in that West syndrome develop from 3-6 months of age and LGS from 1-3 years of age. The same considerations are valid for the early myoclonic encephalopathy (EME). EME seems not to have a specific evolution with age [79].

## Imaging

Imaging studies have substantially improved our understanding of etiology in IS. The hypoxic ischemic encephalopathy is a primary cause in 30% of cases. Delayed myelination seems to be independent of specific etiology [80] and can be present at the onset of cryptogenic West syndrome. Neither the seizure outcome nor developmental status are positively correlated with this finding [81].

Abnormal MRI signal in the basal ganglia due to T8993G mt DNA can indicate a mitocondrial mutation as in three reported case of IS with hypsarrhythmia. Vigabatrin and steroids controlled the spasms. In a follow up of several years the patients were hypotonic and had hyperlactatorrachia. Neuropathy and retinopathy were also present [82]. In four patients with early onset West syndrome a severe hypomyelination and reduction in cerebral white matter has been reported. The patients had profound psychomotor delay, impaired visual attention and acquired microcephaly and spastic tetraplegia [83].

Neuroimaging studies have frequently demonstrated cortical dysplastic lesions. However a cortical dysplasia may not be evident until myelination is advanced and the poor gray-white matter differentiation becomes visible. For these reason the dysplastic lesion can be detected earlier using PET scanning of glucose metabolism. Further study with PET tracers alpha [(11)C] methyl-L-tryptophan and [(11)C] flumazenil as tracers of serotonergic and gabaergic neurotransmitter systems are useful [84]. The absence of PET abnormalities in infants with cryptogenic IS is a good prognostic sign regarding a favourable development or seizure outcome. PET abnormalities can be transient [85] often associated with hypometabolic cortical foci especially in the occipital area. When transient they are not necessary due to structural lesions and do not indicate a poor prognosis [86]. With diffusion tensor imaging it is possible to visualize abnormalities not otherwise demonstrable.

This technique is quite recent and has not been utilized in the patients considered in this review. For more details, see "Progress in epileptic spasms and West syndrome. Guzzetta F, Dalla Bernardina B, Guerrini R, John Libbey Eurotext 2007".

## Prognosis

Outcome in West syndrome varies considerably and is correlated with etiology. In cryptogenic cases prognosis is significantly better than in symptomatic cases. In a study of 45 patients with cryptogenic West syndrome reported by Ducal 30 patients had a favourable outcome. Children with normal neurological history and evaluation before the onset of spasms have a much better prognosis in comparison with children with developmental retardation or abnormal neurologic status. Onset of spasms in the first months of life is also a negative prognostic factor since is related to the etiology. Rapid cessation of spasms and normalization of the EEG are favourable signs [87]. All these aspects are shared by our experience (Fois A: *Umpublished data*).

A successful treatment of West syndrome is a very important endeavour since can change completely the social personal outcome. Opinion regarding this issue are by no means uniform probably reflecting differences related to a retrospective patients evaluation. In a series of 37 patients treated for a long period with high dose tetracosactide depot followed by oral prednisolone slowly tapered for at least five months a normal cognitive outcome was found in 100% of cryptogenic cases treated within one month from the beginning of symptoms [88].

The different outcome of West syndrome depends on the etiology [89]. A short treatment interval from the beginning of the symptoms seem to be associated with a favourable outcome. Reapperance of paroxysmal discharges and evolution to other types of seizures may be associated to undetected lesions [90]. Riikonen has evaluated the long term outcome of 214 Finnish children with West syndrome. A third of the patients died in the follow up 20-35 yars A third of them died before the age of 3 years mostly for infection. Intellectual outcome was normal or slightly impaired in a quarter of the patients. Factors associated with good prognosis were cryptogenic etiology, normal development before treatment, short treatment lag and a good response to ACTH [91].

Infantile spasms may reemerge as intractable epileptic spasms. This events may be very difficult to treat [92]. The risk of a cognitive problems in children with a cryptogenic IS increased when treatment was started after more than three weeks of hypsarrhythmia duration [93].

## Treatment

The best practice to treat IS does not seem to have yet be determined. There is still a lack of consensus about the first choice treatment. Two options are at the moment available: the first is corticotrophin or oral steroids, the other is the use of vigabatrin or other antiepileptic drugs. In selected resistant case a surgical option can also be considered. An international consensus on a therapeutic protocol and standard outcome measures should be adopted. It has also been suggested to abandon the terms cryptogenic and idiopathic using instead symptomatic and nonsymptomatic [94,95].

Sorel first reported in 1958 the beneficial effect of corticotrophin therapy [96]. On later studies modalities of this treatment have been quite different influencing the frequency and the intensity of the adverse events which are mainly related to the dosage and length of treatment. Synthetic derivates seem to be more frequently associated to adverse effects. These include weight and blood pressure changes, infections, also with opportunistic organism, hyperglycemia and electrolyte abnormalities, cushingoid appearance, development of new seizure types, sleep and behaviour abnormalities and decrease of bone density sometimes with fractures, skin rashes with intravascular coagulation and even death. In the referred studies doses of ACTH were however mostly superior to 150 U/square m/day [97,98]. For this reason a much lower dosage and shorter treatment have recently been utilized.

Doses of 3-6 U/kg/day have been administered by Riikonen [99]. Lower dosage has been used in Japan and reported to be as effective as higher doses of previous studies [100]. From 0.2 to 1.28 IU/Kg with a total dosage from 4 to 34.8 IU of synthetic ACTH have been used. The severity of brain volume loss correlated with the daily dosage and total dosage of ACTH. Low dose therapy was effective as the higher doses [101].

Favourable results especially in cryptogenic cases can also obtained with even lower dosage of 0.1 I.U without significant side effects [102]. With 2.5 IU/Kg daily a 77.4% of seizure free patients was obtained but 17% patients encountered severe side effects such as major infections which prompted a stop of the treatment [103]. These results were confirmed by another study with daily doses of 0.96 I.U/Kg for an average of 10 days [104].

Summing up it can be said that when ACTH is used as first line therapy low dosage for short period of treatment seems to be today the best option.

Prednisolone has also been administered. In the United Kingdom Infantile Spasms study Prednisolone and syntetic corticotropin were compared with vigabatrin Oral prednisolone was given at the dosage of 40 mg/day, synthetic corticotropin depot 0.5 mg. on alternate days The results of the two groups of patients were cessation of spasms in 70% of patients treated with corticotropin or prednisolone,54% in patients treated with vigabatrin. Adverse events were reported in 55% of the former group and in 54% of patients treated with vigabatrin [105]. Thyrotropin releasing hormone (TRH) has also been reported to be efficacious in IS Lennox-Gastaut syndrome and myoclonic seizures [106].

Antiepileptic drugs are also used for the treatment of West syndrome. In a trial of combined treatment with benzodiazepines on 24 cases, reduction or cessation of spasms was obtained in 45.8% but the relapse rate was 18.2%. Psychomotor delay was present in 83.4% of cases when treatment was completed. This treatment can be utilized in cases of intolerance or adverse effect with ACTH or vigabatrin [107].

Vigabatrin is used with advantage in treatment of IS especially in cases associated to tuberous sclerosis complex [108-110]. Doses are usually around 100 mg/Kg/day with a gradual increase but maintained at the lowest effective dosage. Superior doses seem not to be of benefit. Vigabatrin is effective and well tolerated in IS. However there is no consensus on how long this treatment should be maintained [111]. This treatment can be associated with reduced visual function [112]. There is no agreement whether this adverse event is related to dosage, length of treatment or if the phenomenon is reversible.

Zonisamide has been reported to be useful in West syndrome. Dosage is variable from 4 to 22 mg/Kg/day. It has been used mainly in Japan. Anorexia, gastrointestinal symptoms and leucopenia are reported as adverse effects. White cell number must be monitored. Good and excellent results are obtained either on spasms and/or hypsarrhythmia [113-115].

Among the more recent antiepileptic drugs, positive allosteric modulators for GABA receptors are effective in controlling infantile spasms. Ganaxolone, a novel neuroactive steroid, has demonstrated outstanding efficacy but is not yet widely available [116,117]. Lamotrigine in low dosage is reported useful in symptomatic IS [118].

There have been limited reports on the beneficial effect of levetiracetam in cryptogenic IS [119-121].

Topiramate, a broad spectrum antiepileptic drug, has been also used recently for treatment of West syndrome in two large studies in China [122,123]. A significant reduction in seizures was obtained. Side effects were somnolence, sedation, lost of appetite and reduced diaphoresis. In the study it seems that in idiopathic patients the results were better than in symptomatic cases [124,125]. Topiramate was started with doses from 1 mg/kg/day and titrated to 20 mg/Kg/day. In all these papers a distinction between results in symptomatic and idiopathic cases is not indicated.

In our experience an add on treatment of vigabatrin 80-100 mg/Kg/day with topiramate 3-3.8 mg/Kg/day obtained a complete clinical and EEG normalization with neurodevelopmental normality which lasted 4 years in 3 patients with idiopathic West syndrome, after gradual withdrawal of both drugs. In one patient with West syndrome associated with TSC there was only a partial improvement [126].

Results with ketogenic diet are considered to be possibly effective or ineffective as an alternative for the treatment of IS [127,128].

The identification of focal or multifocal cortical lesions with MRI and PET (positron emission tomography) has led to the possibility of surgical approach for some patients with intractable IS. When a single lesion is present on MRI and/or PET and there is a correlation with EEG localization, the results of surgical treatment is reported to be quite favourable. [(11)c] Flumazenil (FM2) which labels GABA (A) receptors and alpha [(11)c] methyl-l-tryptophan (AMT) which is a capable of differentiating between epileptogenic and non-epileptogenic lesions, can be particulary useful for this type of evaluation [129,130].

When there are no localization, favourable results have been reported in two patients with intractable IS preceded by partial seizures with multiple subpial transections (MST) [131].

Iv immunoglobulins can be used as coadjuvant treatment in cases which are difficult to control even in symptomatic cases [132] and also for juvenile epileptic spasms [133]. Prospective clinical trials however are necessary in order to evaluate the usefulness of this treatment.

High dose Vitamin B6 (pyridoxine) are reported to be effective in West syndrome. The response to treatment is rapid and seizures disappear within the first 2 weeks of treatment. Gastrointestinal symptoms and liver dysfunctions are observed in 40-70% of subjects but they resolve after reduction of dosage or discontinuation [134].

Pyridoxal phosphate can be effective when there is no response to pyridoxine [135,136].

## Suggested Diagnostic Approach and Treatment

In order to give some practical cues we indicate in the hereafter what we believe should be the approach when dealing with a patient with IS.

With a careful clinical and developmental history and examination, as well as the laboratory and instrumental evaluation that are indicated in every single case, it is necessary to distinguish between symptomatic and idiopathic IS. In particular the MRI must be of a very good quality in order to disclose even minimal structural abnormalities.

This workup must be performed before administering any type of therapy

In idiopathic IS it is wise to begin the treatment with the administration of 300 mg. of pyridoxine per os or i.m. for 3 days. If there is no clinical or EEG response Vigabatrin (VGB) and and topiramate (TPM) are preferred to ACTH, because side effects of these two drugs are considered to be less dangerous then the those caused by corticotropin treatment.

**Figure 2:**
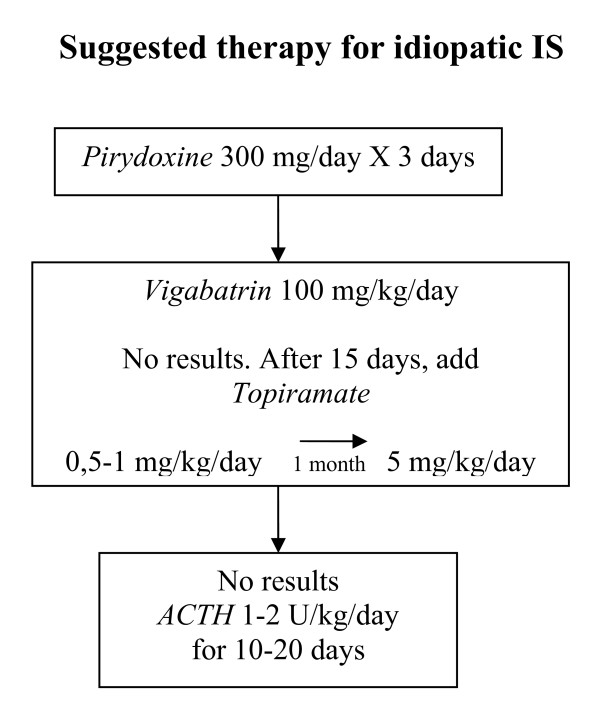
**Flow chart for treatment of infantile spasms**. See details in text.

VGB is titrated to 100 mg./kg/day in 7 days period. If after 15 days of treatment no results are obtained TPM 0.5 -1 mg./kg./day is prescribed and titrated to 5 mg./kg, up to 5 mg./kg in a month if necessary. If there is no response to this treatment the use of Synachten depot is considered with a dosage of 1-2 U./kg. day for a maximum of 20 days. A positive response must be obtained within this period with disappearance of the spasms and normalization of the EEG. These suggestions are summarized in Fig. 2.

A clinical and EEG evaluation must be programmed every month for the following six months. If the clinical and EEG situation is normal at this date a gradual decrease of the TPM dose in a three month period is indicated. VGB thereafter can be also in the same time period tapered off, thus minimizing the adverse effects of this drug on the visual field. Of course the EEG must always remain normal. Clinical and EEG evaluation must then be programmed every 3 months for one year.

## Symptomatic IS

It s not clear which therapy is recommended in symptomatic IS. When the etiology is TSC, VGB possibly associated to TPM, should be considered. Frequently spasms disappear to be substituted with partial seizures with or without generalization. Zonisamide, lamotrigine, and levetiracetam as well as other AEDs can also be used.

In other symptomatic IS it is necessary to evaluate the aetiology and prescribe the therapy that is considered more effective.

It is however recommended that therapy be planned according to aetiology as in any other epileptic condition. Surgical treatment can be an option when focal lesions are demonstrated.

## Conclusions

IS is an age dependent epileptic encephalopathy associated to different aetiologies which are today identifiable in the majority of cases.

Idiopathic IS is still an etiologically unresolved problem. Therapeutic response to corticotrophin and steroids might suggest an immunological pathogenesis.

However, in apparently similar idiopathic cases complete results can be obtained with AEDs which do not have a demonstrated activity on the immune system.

In any case from a practical point of view in is important to stress that a fast and long lasting normalization can be obtained in idiopathic cases with early diagnosis and proper therapy.

## Competing interests

The author declares that they have no competing interests.

## Appendix 1

### Pathologies Associated with Infantile Spasms

#### Metabolic Disorders

Alexander disease

biotinidase deficiency

CDG deficient syndrome

d-bifunctional protein deficiency

d-glyceric aciduria

Hurler syndrome

hypoglycaemia

Leigh syndrome

Menkes disease

methylmalonic aciduria

mitochondrial diseases

phenylketonuria

propionic acidemia

pyruvate dehydrogenase E1 alpha subunit deficiency

SCAD deficiency

vitamin B12 deficiency

#### Malformations and genetic disorders

cri du chat syndrome

Down's syndrome

Familiar periventricular heterotopia

Gyration abnormalities

malformation of cortical development

microcephaly, lissencephaly and tetralogy of Fallot

Neurofibromatosis

polymicrogiria

sodium channel mutations

terminal 1p36 deletion

trisomy 4 p

Tuberous sclerosis complex

#### Dysmorphogenetic sindromes

Aicardi syndrome

Kabuki syndrome

Norrie disease

Schinzel-Giedion syndrome

Sotos syndrome

Williams syndrome with craniosynostosis

### Other Conditions

Cerebral tuberculomas and neoplasms

Cytomegalovirus

DEND syndrome

Encephalomalacia

PEHO syndrome

Sturge-Weber syndrome

### Familiar Cases

ARX mutations

CDKL5 mutations?
